# Achievements and problems of psychosocial rehabilitation: Results of sociological research in the volga federal district

**DOI:** 10.1192/j.eurpsy.2021.1341

**Published:** 2021-08-13

**Authors:** V. Mitikhin, T. Solokhina, G. Tiumenkova, V. Yastrebova

**Affiliations:** Department Of Mental Health Support Systems Research Centre, Mental Health Research Centre, Moscow, Russian Federation

**Keywords:** sociological, psychosocial interventions, effectiveness, Psychosocial rehabilitation

## Abstract

**Introduction:**

The active development of psychosocial rehabilitation (PSR) has been taking place in Russia within latest two decades. In this regard, analysis of the accumulated experience and problems’ identification in the PSR field is relevant.

**Objectives:**

Conducting a sociological study in the Volga Federal District (VFD) to work out measures for further PSR system development.

**Methods:**

Sociological, statistical, original semi-structured questionnaire on PSR application, including 26 questions.

**Results:**

63 institutions providing psychiatric care in 14 large regions of the VFD participated in the study. Achievements in the field of PSR include: introduction of new forms of rehabilitation care, modern psychosocial interventions; development of the volunteer sector and others. A number of systemic problems were also identified: more pronounced decrease in the availability of psychiatrists in VFD compared to the Russian Federation (RF) as a whole (in the VFD 0.76 psychiatrists per 10 thousand population in 2017 and 0.74 per 10 thousand population in 2018; in RF: 0.83 psychiatrists per 10 thousand population in 2017, 0.82 per 10 thousand in 2018); insufficient provision with psychotherapists, psychologists, social workers, which varies considerably in different territories (up to 10 times); insufficient use of non-profit organizations’ (NPOs) potential; lack of a unified system for assessing PSR effectiveness.

**Conclusions:**

Measures for development of PSR were proposed: improving staffing levels and qualifications of employees, introducing psychosocial interventions with proven effectiveness; dissemination of successful experience of NPOs, development of methodological tools for assessing effectiveness of PSR, its standardization and others.

